# Current Animal Models for Understanding the Pathology Caused by the Respiratory Syncytial Virus

**DOI:** 10.3389/fmicb.2019.00873

**Published:** 2019-05-03

**Authors:** María José Altamirano-Lagos, Fabián E. Díaz, Miguel Andrés Mansilla, Daniela Rivera-Pérez, Daniel Soto, Jodi L. McGill, Abel E. Vasquez, Alexis M. Kalergis

**Affiliations:** ^1^ Departamento de Genética Molecular y Microbiología, Facultad de Ciencias Biológicas, Millennium Institute on Immunology and Immunotherapy, Pontificia Universidad Católica de Chile, Santiago, Chile; ^2^ Sección Biotecnología, Instituto de Salud Pública de Chile, Santiago, Chile; ^3^ Department of Veterinary Microbiology and Preventive Medicine, Iowa State University, Ames, IA, United States; ^4^ Facultad de Medicina y Ciencia, Universidad San Sebastián, Providencia, Santiago, Chile; ^5^ Departamento de Endocrinología, Facultad de Medicina, Pontificia Universidad Católica de Chile, Santiago, Chile

**Keywords:** human respiratory syncytial virus, bovine respiratory syncytial virus, lower respiratory tract infections, rodent model, non-human primate model

## Abstract

The human respiratory syncytial virus (hRSV) is the main etiologic agent of severe lower respiratory tract infections that affect young children throughout the world, associated with significant morbidity and mortality, becoming a serious public health problem globally. Up to date, no licensed vaccines are available to prevent severe hRSV-induced disease, and the generation of safe-effective vaccines has been a challenging task, requiring constant biomedical research aimed to overcome this ailment. Among the difficulties presented by the study of this pathogen, it arises the fact that there is no single animal model that resembles all aspects of the human pathology, which is due to the specificity that this pathogen has for the human host. Thus, for the study of hRSV, different animal models might be employed, depending on the goal of the study. Of all the existing models, the murine model has been the most frequent model of choice for biomedical studies worldwide and has been of great importance at contributing to the development and understanding of vaccines and therapies against hRSV. The most notable use of the murine model is that it is very useful as a first approach in the development of vaccines or therapies such as monoclonal antibodies, suggesting in this way the direction that research could have in other preclinical models that have higher maintenance costs and more complex requirements in its management. However, several additional different models for studying hRSV, such as other rodents, mustelids, ruminants, and non-human primates, have been explored, offering advantages over the murine model. In this review, we discuss the various applications of animal models to the study of hRSV-induced disease and the advantages and disadvantages of each model, highlighting the potential of each model to elucidate different features of the pathology caused by the hRSV infection.

## Introduction

Human respiratory syncytial virus (hRSV) is an enveloped, single-stranded, and negative-sense RNA virus belonging to order *Mononegavirales*, family *Pneumoviridae*, genus *Orthopneumovirus*, species *Human orthopneumovirus* (Hacking and Hull, 2002; Borchers et al., 2013; Afonso et al., 2016; Snoeck et al., 2018). This virus is a human pathogen that causes a major burden in public health, both in developing and in industrialized countries (Simoes, 2003; Zang et al., 2015; Kuhdari et al., 2018). Noteworthy, hRSV is the leading cause of acute respiratory infection in newborns and of severe lower tract respiratory disease (LTRD) in children, with an estimation of 33.8 million of RSV-associated acute LTRD episodes in children less than 5 years old in 2005 (Nair et al., 2010). Estimations indicate that this virus is responsible for up to 3.4 million of hospital admission due to severe acute LTRD (Nair et al., 2010) and constitutes the leading cause of acute bronchiolitis and subsequent hospital admissions in industrialized countries (Bush and Thomson, 2007). Importantly, this virus is an important cause of mortality in young children in developing countries. In 2015, it was estimated that 59,600 hospitalized infants younger than 5 years old have died from hRSV-related LTRD worldwide (Shi et al., 2017; Scheltema et al., 2018).

Several attempts to develop safe and protective vaccines for the high-risk groups have been ineffective, and currently, there is no licensed vaccine for this pathogen (Hurwitz, 2011). Therefore, there is an urgent need for the development of a hRSV vaccine. In addition, the efficacy of the single licensed therapeutic option remains controversial, raising interest in the development of alternative therapeutic approaches against this pathogen (Canziani et al., 2012; Ispas et al., 2015; Muñoz-Durango et al., 2018; Simon et al., 2018). Therefore, the implementation of functional animal models for studying this virus has emerged as a critical and indispensable aspect underlying the development of immunotherapies and vaccines against hRSV (Hurwitz, 2011). For this reason, the development of different animal models for studying several aspects of hRSV has been very important and is still a field where research is focused. Since no animal model reflects all aspects of this viral infection and disease (Taylor, 2017), several models have been used in the study of hRSV, ranging from rodents and small mammals to large animals and non-human primates. This results from high specificity of hRSV for the human host, lacking an animal reservoir in nature (Collins and Graham, 2008). This feature has greatly hindered the development of an exclusive animal model, and therefore, the choice of the more suitable animal model required for each researcher will depend strongly on the aspect of the infection that needs to be studied and the investigative hypothesis proposed (Jorquera et al., 2016). The most commonly used animals have been rodents, such as mice (Graham et al., 1988; Bueno et al., 2008) and cotton rats (Prince et al., 1978, 1983; Sawada and Nakayama, 2016); ruminants (Elvander, 1996; Woolums et al., 1999, 2004; Meyerholz et al., 2004; Derscheid and Ackermann, 2012; Ackermann, 2014); and non-human primates (Kakuk et al., 1993; Szentiks et al., 2009), but at the present, the diversification of animal models is a requirement for addressing the diverse problematics of this viral infection and the development of vaccines and treatments. For this reason, the objective of this article is to review the several animal models used and their applications and to discuss their pros and cons. Finally, and based on the current information, recommendations of use are made, besides highlighting the role of the use of the murine model as a first approximation in the recent preclinical studies of new vaccines and antiviral treatments.

## Non-Human Primate Models of hRSV

Mainly with the objective of vaccine efficacy and safety testing, several species of non-human primates (NHPs) have been used as animal models for hRSV infection, including chimpanzees (*Pan troglodytes*) (Szentiks et al., 2009), African green monkeys (*Chlorocebus sabaeus*), macaques (*Macaca* spp.) (Simoes et al., 1999; De Swart et al., 2002; Patton et al., 2015), and owl monkeys (*Aotus* spp.) (Prince et al., 1979). This group of species shares several anatomic and physiologic similarities to humans, but its application as an animal model of infection is challenging due to economical, technical, and ethical issues. Only chimpanzees are fully permissive to hRSV infection (Belshe et al., 1977; Crowe et al., 1993; Teng et al., 2000) and develop almost exclusively upper respiratory tract disease (URTD) symptoms in experimental studies.

### The Chimpanzee

hRSV was isolated for the first time not from humans, but from a group of chimpanzees naturally infected by the virus, which displayed signs of URTD (Morris et al., 1956). The experimental infection on this species has shown varying results, but high viral replication has been reported with viral doses lower than 10^4^ plaque-forming units (PFUs; Belshe et al., 1977). Viral replication measured in nasopharyngeal and tracheal samples indicated that peak viral loads reached were 1–2 order of magnitude higher than the initial inoculum, and persistence of virus was shown to last 6–10 days post-infection (DPI; Belshe et al., 1977; Crowe et al., 1993; Teng et al., 2000). Although infected chimpanzees show clear URTD signs (Blount et al., 1956), no low respiratory tract disease (LTRD) has been reported under experimental conditions; therefore, human clinical disease condition is not completely replicated in this species. However, naturally infected chimpanzees with fatal LTRD have been reported, including viral antigen presence in lungs and histopathological changes including high neutrophil infiltration, edema, and the deposition of hyaline membranes (Belshe et al., 1977; Szentiks et al., 2009; Unwin et al., 2013). Natural co-infections with *Streptococcus pneumoniae* have also been reported and may be enhancing those naturally occurring infections (Szentiks et al., 2009; Nguyen et al., 2015).

Applications of this NHP model have included the evaluation of protective efficacy and safety of live-attenuated and recombinant vaccine candidates (Richardson et al., 1978; Collins et al., 1990; Crowe et al., 1993; Teng et al., 2000). Similar body temperatures between chimpanzees and humans have allowed studies with candidate temperature-sensitive vaccines (Council, 2011). Vaccine candidates consisting of the recombinant expression of F and G glycoproteins by modified vaccinia viruses have shown to induce low levels of neutralizing antibodies (NAs) and protection to viral challenge in chimpanzees (Collins et al., 1990; Crowe et al., 1993). On the contrary, temperature-sensitive mutant hRSV vaccines that are highly restricted at replication but highly immunogenic in chimpanzees have induced protective responses against hRSV infection in this species, although protective mechanisms have not been defined in detail (Richardson et al., 1978; Crowe et al., 1995). Permissive replication of hRSV is the most remarkable advantage of this model, being the unique species reflecting the viral replication in humans (Belshe et al., 1977; Crowe et al., 1993). Anatomical, physiological, and genetic similarities also represent a unique advantage of the use of this model.

However, several disadvantages and limitations apply to this model, and the appearance of several other useful models has diminished the utilization of chimpanzees. The economical, logistical, and ethical cost of research on chimpanzees is extremely high, and therefore, this NHP is not commonly available to research groups (Tardif et al., 2013). Published studies to date mostly yielded inconclusive results due to small sample sizes and genetic heterogeneity of animals. Lack of inbred strains, immunological tools, and reagents are also an important restriction to the use of this model (Bem et al., 2011; Jorquera et al., 2016). Finally, specialized housing, handling, and caring of this species are mandatory (Tardif et al., 2013).

The National Research Council has established that this model may be necessary exclusively for testing the candidate vaccines, based on the suitability of this model to serve as surrogate model for seronegative infants (Council, 2011).

### The Macaque

Permissiveness of species within *Macaca* spp. to hRSV infection is low, with high dose intranasal (IN) or intratracheal (IT) inocula reaching low or moderate viral replication capacity, despite the age of individuals (Simoes et al., 1999; De Swart et al., 2002; McArthur-Vaughan and Gershwin, 2002; Grunwald et al., 2014; Grandin et al., 2015). Although clinical disease is rare, a report has shown evidence for mild respiratory disease with fever and increased respiratory rate in juvenile rhesus macaques associated to bronchiolitis, bronchitis, and interstitial pneumonia (McArthur-Vaughan and Gershwin, 2002). The virus has been re-isolated from lungs but at low viral titers (Simoes et al., 1999; McArthur-Vaughan and Gershwin, 2002). Studies using the formalin-inactivated hRSV vaccine (FI-hRSV) have shown enhanced disease with cellular peribronchiolar, perivascular infiltration, and high viral titers, but that enhanced response might be affected by immune responses to non-viral antigens present in the viral challenge preparations (Ponnuraj et al., 2001; De Swart et al., 2002). A vaccine candidate based on the Fusion (F) protein of hRSV adjuvanted with GLA-SE was evaluated in cynomolgus macaques and induced a robust humoral and Th1-biased cellular immunity (Patton et al., 2015). Further, lack of protection and evidence of enhanced disease were observed in infant macaques after immunization with a modified vaccinia virus Ankara vector expressing the hRSV F and G proteins (de Waal et al., 2004). Also, a heterologous adenovirus vector vaccine with the F subunit of hRSV have been evaluated in rhesus macaques (Grunwald et al., 2014). A mucosal booster immunization showed to be effective at reducing viral replication, with an immune response that included a high and persistent expansion of CD4^+^ and CD8^+^ T cells in the lower respiratory tract mucosal sites. These data suggest that this vaccine scheme is effective for eliciting protective immune responses at mucosal effector sites in this animal species (Grunwald et al., 2014). The pros and cons for using this animal model are equivalent to those previously mentioned for chimpanzees.

### The African Green Monkey

African green monkeys (AGMs) are less susceptible to hRSV infection than chimpanzees (Kakuk et al., 1993), often lack clinical manifestations after hRSV infection, and develop only minor histopathological changes (Kakuk et al., 1993; Ispas et al., 2015). Conversely, the viral load constitutes a more reliable disease parameter in this animal model (Kakuk et al., 1993; Eyles et al., 2013; Ispas et al., 2015). Mild signs, including rhinorrhea, coughing, sneezing, and wheezing, have been described in AGMs following IN and IT administration of the hRSV A2 strain AGMs. Further, viral shedding in the oropharynx has been detected for 8 days in these animals (Kakuk et al., 1993). Studies on AGMs have focused on vaccine candidate testing, including recombinant vaccines (Kakuk et al., 1993; Tang et al., 2004; Jones et al., 2012; Wang et al., 2017) and adjuvanted subunit vaccines (Eyles et al., 2013). Vaccines that had shown efficacy in rodent challenge studies have failed to elicit appropriate responses in AGMs, which could be due to the immunological response itself or to the different experimental design relative to the vaccination and challenge (Taylor, 2017). These observations underscore the difficulty of translating results from rodent species to primates (Eyles et al., 2013). Additionally, antiviral effects of TMC353121, a fusion inhibitor, have been successfully tested in AGMs as a preclinical model (Ispas et al., 2015). The first primate model for FI-hRSV-induced enhanced respiratory disease was developed on this species, with airway and parenchymal inflammatory changes in the vaccinated animals, which could not be evidenced by viral titers (Kakuk et al., 1993). Although less expensive than chimpanzees, utilization of this species has most of the general disadvantages in which NHP models display, as those previously discussed.

### The Owl Monkey

Only a few studies have explored the use of this species as an animal model for hRSV infection (Prince et al., 1979; Koff et al., 1983; Hemming et al., 1995). Prince and collaborators have reported that an IN inoculation of 10^4^ PFU of hRSV can result in high viral titers, rhinorrhea, and the development of low levels of neutralizing antibody (NA) virus titers appearing at 14 days post-infection and reaching a peak at 28 days post-infection. Viral infection was observed until 8 to 17 days (Prince et al., 1979; Koff et al., 1983). Antibody-dependent cellular cytotoxicity has been studied in owl monkeys against human epithelial HEp-2 cells infected with hRSV, with peripheral mononuclear blood cells in the presence of hRSV antibodies causing lysis of infected HEp-2 cells in an *in vitro* assay (Koff et al., 1983). Reduction of viral shedding from nose and trachea has been reported after intravenous administration of human immunoglobulin (IVIG) in owl monkeys that previously received an intratracheal (IT) hRSV challenge (Hemming et al., 1995). Response to vaccination in this species has been scarcely studied; a single dose of recombinant vaccinia virus expressing the F or G glycoprotein of hRSV has been reported to be effective in controlling hRSV-induced LRT (Olmsted et al., 1988).

## Rodent Models for hRSV Disease

The order *Rodentia* includes a variety of small mammals that have been widely used as models of human pathogens in immunological and infection studies, including vaccine testing studies, and elucidation of immunopathogenic mechanisms (Andersen and Winter, 2017). Despite differences at the infective cycle developed by hRSV infection, permissiveness for viral replication, and host immune system differences between mice and humans, the laboratory mouse model remains to date as one of the most prevalent animal models used in hRSV studies. However, the use of the cotton rat has allowed the modeling of several aspects on this infection that the mouse model fails to resemble, including viral replication permissiveness, which is improved in cotton rats. These two species are systematically used nowadays and have allowed researchers to make significant progress in vaccine and immunotherapy development. Further, other rodent models have also been utilized, as described later.

### The Mouse Model

The laboratory mouse (*Mus musculus*) is the most frequently used animal as a model in the field of biomedical sciences and is the model of choice in immunological and infection research, as well as for the evaluation of vaccines and therapies worldwide (Lee et al., 2012; Ehret et al., 2017). Although the development of vaccines and new therapeutic approaches against hRSV has been a challenge for scientists to date (Salazar et al., 2017; Mazur et al., 2018), mouse models have been very important to achieve past and recent advances in preclinical tests of new vaccines and antiviral treatments. Further, these models have contributed significantly to elucidate their mechanisms of action (Hurwitz, 2011; Heylen et al., 2017). Noteworthy, the clinical symptomatology of the hRSV disease after mouse infection has certain differences when compared to human disease. Besides, clinical differences are also observed between different strains of mice (Openshaw and Tregoning, 2005). Another feature of the murine model for hRSV infection is that these animals must be challenged with large doses (~10^7^ PFU) to detect viral replication in the lungs (Graham et al., 1988). Among mice strains available, the BALB/c mouse is commonly used to study the immunopathology caused by hRSV infection, since it is semi-permissive to lung viral replication. The disease manifests with piloerection, reduced activity, and weight loss (Graham et al., 1988; Kong et al., 2005). Furthermore, moderate bronchiolitis is observed in these mice (Bueno et al., 2008). Importantly, loss of body weight within the first 3 days of infection and infiltration of lungs with neutrophils are two well-established disease parameters to assess protection against this serious disease (Bueno et al., 2008).

It has been reported that cell-mediated immunity decreases as individuals get older. Therefore, studying CD8^+^ T-lymphocyte populations in different age groups is a relevant topic to address. (Fulton et al., 2013; Mosquera et al., 2014). The aged BALB/c mice generate a weak primary response of CD8^+^ T cells specific for hRSV in the lung, which is associated with a delay in viral clearance. When evaluating the maximum magnitudes of the responses of virus-specific CD8^+^ T cells in lung and airways, significant decreases have been seen in aged BALB/c mice when compared with young BALB/c mice (Fulton et al., 2013). Infected aged BALB/c mice develop severe inflammatory changes with a predominance of neutrophils and lymphocytes, in addition to diffuse alveolar damage 4 days post-infection (Mosquera et al., 2014). It has been shown that both the kinetics and the magnitude of antiviral gene expression decrease as a result of advanced age. In addition to the delay in cytokine signaling and the induction of the receptor pattern recognition, it has been found that TLR 7/8 signaling is altered in alveolar macrophages in elderly mice, and this shows that there are inherent differences in response to hRSV infection in pneumonia models of young BALB/c mice versus the model of aged BALB/c mice (Wong et al., 2014). Experimentally, it has been described that a senescence prone SAM-P1 mouse strain (H-2 K), which shares the genetic background of AKR/J mice, is more susceptible to infection by hRSV, and shows a deficient CD8^+^ T cell response, as well as a lower gamma-interferon (INF-γ) production, which contrasts to high interleukin (IL)-4 production (Liu and Kimura, 2007). Noteworthy, this animal model allows for the generation of humanized mice or mice with a human immune system (HIS mice; Sharma et al., 2016), which has been also used to evaluate vaccine efficacy (Sharma et al., 2016). During the first 3 days of infection, the HIS mice lost more weight and eliminated hRSV faster than did NOD-scid gamma mice (NSG mice). These mice were generated by the introduction of an adeno-associated virus serotype 9 (AAV9) vector carrying human cytokine genes into highly immunodeficient NOD-scid gamma (NSG). The pathological characteristics induced by hRSV infection in HIS mice include peribronchiolar inflammation, neutrophil predominance in the bronchoalveolar lavage fluid, and enhanced production of mucus in the respiratory tract (Sharma et al., 2016). In addition, the mouse model has been extensively used to perform preclinical studies for vaccine and therapies for this virus (Hurwitz, 2011). Recently, vaccine efficacy studies were performed in mice to evaluate safety and immunogenicity of a vaccine consisting of a recombinant strain of bacillus Calmette-Guérin (rBCG) expressing the nucleoprotein antigen of hRSV (Cautivo et al., 2010; Céspedes et al., 2017). A single immunization with the rBCG vaccine elicited protective Th1 type immunity against hRSV in mice, preventing weight loss due to infection, as well as reducing viral replication and disease severity in these animals (Céspedes et al., 2017). Vaccination with rBCG promotes the elimination of the virus at the pulmonary level and prevents development of interstitial pneumonia without evident adverse effects (Céspedes et al., 2017). Moreover, subunit vaccines against hRSV based on a novel genome replication-deficient Sendai virus (SeV) vector expressing the F protein as a genetically stable antigen have been studied (Wiegand et al., 2017). After IN or intramuscular (IM) immunization of BALB/c mice, a robust hRSV-specific immune response was induced, consisting of serum IgG and neutralizing antibodies, as well as cytotoxic T cells. Further, IN immunization was also able to stimulate hRSV-specific mucosal IgA in the upper and lower respiratory tract of these animals (Wiegand et al., 2017). The mouse is also a useful model for the study of basic aspects of mechanisms related to the pathogenesis of asthma as a consequence of hRSV infection (Peebles and Graham, 2005). It is well established that the mouse model has multiple comparative advantages in relation to other animals, such as genetic homogeneity between consanguineous animals; availability of humanized, knockout, and transgenic stains; very good reproductive performance; ease of maintenance in confined spaces; non-complex nutritional requirements; and wide variety of reagents to study the immune response. Additionally, the similarities between the mouse and human immunological response, as well as the docile nature of the laboratory mice and non-complex management, make this species one of the most used for studies relative to infections and immunity worldwide (Mestas and Hughes, 2004; Drake, 2013; Sharma et al., 2016).

### The Cotton Rat Model

The cotton rat (*Sigmodon hispidus*) is currently among the most extensively used animal models of hRSV and other human infectious respiratory diseases, in part due to the semi-permissive replication of several respiratory viral agents and the developing industry of genetic and immunological tools applicable on this model. Up to date, this model has been successfully used for several studies in hRSV, including antibody prophylaxis (Ottolini et al., 2002), vaccine testing (Widjojoatmodjo et al., 2010; Garlapati et al., 2012; Kamphuis et al., 2013; Rostad et al., 2016; Stobart et al., 2016; Widjaja et al., 2016; Fuentes et al., 2017), FI-RSV enhanced respiratory disease (Prince et al., 1986; Sawada and Nakayama, 2016; Widjaja et al., 2016), maternally induced immunity (Prince et al., 1983; Blanco et al., 2015), and susceptibility of high-risk human groups (Boukhvalova and Blanco, 2013). In fact, the main advantage of this model is its greater permissiveness to hRSV infection than inbred mice, other rodents, and animals in general, reaching a replication that is 50–1,000 fold higher than mouse strains (Prince et al., 1979). Infection with hRSV undergoes active replication in lungs and nasal tissue, lasting about 6–9 days, respectively. However, tracheal replication reaches only low titers (Prince et al., 1978). Histopathological examinations have revealed mild lesions as early as day 2, including bronchitis, bronchiolitis, and exudative rhinitis (Prince et al., 1978). Interestingly, with higher doses (10^6^ PFU), cotton rats develop alveolitis, peribronchiolitis, and interstitial pneumonitis, therefore reflecting a direct relationship between viral replication, histopathology, and the infectious dose inoculated (Prince et al., 1978). Although the quality and timing of pathological changes observed in cotton rats after hRSV infection share several features with the natural human hRSV infection, bronchiolitis is notoriously less severe in cotton rats (Prince et al., 1978, 1986). The absence of clinical signs, such as cough, rhinorrhea, and fever, constitutes a disadvantage that should be considered for certain studies. Although cotton rats remain susceptible through the whole life, viral replication and persistence are major in infant animals. Reinfection of this species leads to an inflammatory response in lungs that is characterized by the absence of viral production due to an abortive viral replication (Prince et al., 1979; Boukhvalova et al., 2007a), which is paralleled by an early upregulation of interferon response and expression of interferon-inducible MX genes (Pletneva et al., 2008). The presence of MX genes is noteworthy, since Mx1 and Mx2 are functionally absent in inbred mouse strains C57BL/6, BALB/c, and CBA/J, and is an important component of the antiviral innate response (Staeheli et al., 1993). In terms of the immune response to experimental infection, the arachidonic acid pathway is upregulated after cotton rat infection with hRSV and has been associated to the severity of disease (Richardson et al., 2005). Cytokine synthesis in lung after hRSV infection reveals an increase in IFN-γ, IL-10, IL-6, CCL-2, and growth-regulated oncogene, reaching a peak at day 4 after infection in infant cotton rats, which is delayed to day 6 in aged individuals (Boukhvalova et al., 2007b).

Response to repetitive exposures to hRSV has been evaluated in cotton rats, showing that during the first infection there is an absence of neutralizing antibody production, which results in a lack of protection to a second infection. During a second infection, a neutralizing antibody response and a CD8^+^IFN-*γ*^+^ T cell response take place, which seem to be nevertheless unable to clear the virus from the lungs (Yamaji et al., 2016).

The study of maternal transferred immunity is one of the earliest applications for this model (Blanco et al., 2015). Both colostrum feeding and transplacental immunity can provide protection in lungs and nasal tissue in infant cotton rats gestated by immunized mothers challenged with hRSV prior to litter birth. Unfortunately, this passively transferred protection is transient and significantly reduced after 4 weeks from birth (Prince et al., 1983; Blanco et al., 2015). This protective effect is correlated to serum neutralizing antibodies, with titers >1:380 being protective in infant cotton rats, which is similar to human infant passively-acquired immunity, which results in reduced susceptibility to infection with antibody titers equal or higher to 1:400 (Prince et al., 1983, 1985). However, the lack of correlation in some studied animals suggests that other immune mediators might be contributing to maternally transmitted protective immunity (Prince et al., 1983). Interestingly, a strong correlation between hRSV-specific neutralizing antibody titers in cotton rat mothers and their litters has been described, and the protection provided by those antibodies appears to be inversely associated to cytokine expression in lungs (Blanco et al., 2015). Also, the maternally acquired humoral immunity is responsible for suppressing vaccine immunogenicity in infant cotton rats (Prince et al., 1979, 1982), thereby highlighting the difficulties of developing a vaccine for human infants.

The mentioned correlation between antibody levels as a way of providing protection in cotton rats and human infants led to several studies (Saez-Llorens et al., 1998; Johnson et al., 1999), and to the development of a commercial antibody formulation for prophylactic use in human infants in high risk of severe hRSV disease (Olchanski et al., 2018). The ability of predicting the success of immunoprophylaxis in infants and of assessing the dose of antibody required to elicit protective serum levels is one of the most remarkable milestones of this animal model (Prince et al., 1985; Group, 1998). It is important to mention that those studies advanced to clinical phases without the need of intermediate studies in NHPs and are currently available from commercial use in patients (Niewiesk and Prince, 2002). On the other hand, research on immunotherapeutic use of antibodies against hRSV has been conducted, but has yielded dissimilar results. Several antibodies tested have been useful for controlling viral replication but not for ameliorating lung pathology and disease severity (Rodriguez et al., 1997; Malley et al., 1998; Prince et al., 2000). More recently, a novel Ig formulation, RI-002, was successfully tested in cotton rats for treating immunocompromised patients. This formulation, containing high quantities of hRSV-specific neutralizing antibodies, inhibited the prolonged hRSV replication, which is typical in immunocompromised rats (Johnson et al., 1982) and reduced dissemination of replicative virus. Pulmonary interstitial inflammation and epithelial hyperplasia were also reduced by RI-002 administration, therefore providing evidence of a beneficial therapy for hRSV disease outcome in immunocompromised cotton rats (Boukhvalova et al., 2016) results that should be further investigated on its ability to reduce disease symptoms in humans.

Research on the pathology of vaccine-enhanced respiratory disease, described decades ago in human infants, also finds a suitable model in cotton rats. Knowledge on the mechanisms of this manifestation is critical for future studies on vaccine development. FI-hRSV vaccinated cotton rats subsequently challenged with hRSV present an increased pulmonary pathology, with severe alveolitis as a hallmark of histopathological changes, as well as a rise in neutrophil and lymphocyte infiltration (Prince et al., 1986). Additionally, association of Th2 polarization as a known mechanism of vaccine-enhanced respiratory disease has been studied in this model in which an upregulation of Th2 cytokines (IL-4, IL-10, IL-13, and CCL5) has been observed (Boukhvalova et al., 2006; Sawada and Nakayama, 2016). However, an increase in Th1 chemokines and cytokines has also been reported, and it has been suggested that those pro-inflammatory cytokines and chemokines may play a role in enhanced pulmonary inflammation (Prince et al., 2001; Boukhvalova et al., 2006). Immunodominance of post-fusion-specific site I of F protein elicits weak neutralizing antibody responses against that site in FI-RSV-immunized cotton rats and an absence of an efficient neutralizing response by other antibodies (Widjaja et al., 2016). This situation contrasts with immunization response to experimentally infected or vaccinated cotton rats, which showed a high virus neutralizing capacity from pre-fusion specific antibodies binding antigenic site Ø and other parts of the F protein, and may account for the mechanism on FI-hRSV-enhanced respiratory disease (Widjaja et al., 2016). Noteworthy, limitations of some studies performed in cotton rats have been revealed, including observations on the role of non-viral products in vaccine or challenge inoculated medium as drivers of alveolitis, which appears to be mediated chiefly by T cell specific responses to non-viral antigens, having the hRSV antigens a limited role as co-factors in the inflammation and disease manifestation (Shaw et al., 2013).

Despite limitations, cotton rats have been the main model in research on development of efficient and secure vaccine candidates, and several vaccine candidates have been recently tested in this model including subunit vaccines (Garlapati et al., 2012), recombinant vaccines (Widjojoatmodjo et al., 2010, 2015; Stobart et al., 2016; Fuentes et al., 2017), VLPs and virosomes with viral subunits (Kamphuis et al., 2013; Cullen et al., 2015), as well as live attenuated vaccines (Luongo et al., 2013; Stobart et al., 2016), therefore confirming the relevance of this animal model for those studies. However, prediction capacity of this model on protective responses after vaccination in humans is still unknown, and further investigations are required to elucidate this question.

Increased susceptibility to severe hRSV infection occurring in immunosuppressed infants and older people has been successfully modeled in the cotton rat model. Late clearance of virus is classical in immunosuppressed cotton rats, therefore impacting in disease pathogenesis (Ottolini et al., 1999). Also, this model has been used to test prophylactic and therapeutic schemes in immunosuppressed rats, reflecting the need of multiple administrations of Ig to diminish viral replication (Ottolini et al., 1999; Boukhvalova et al., 2016). Unlike mice, infant cotton rats permit a greater viral replication with a greater persistence in URT than 4-week-age rats and mount a less efficient NA response than adult animals (Prince et al., 1978). In elderly, immunosenescent cotton rats (>6-month age), prolonged viral persistence is present (Curtis et al., 2002), and peak expression of cytokines is delayed in older rats (>6 month age) than young age rats (<2-month age) (Boukhvalova et al., 2007b).

Technical advantages on the use of this model include the availability of commercial and laboratory-owned inbred strains, as well as the increasing quantity of immunological reagents and assays, which, added to similarities between cotton rats and human innate immune system (Boukhvalova and Blanco, 2013), make this species an interesting and useful model for pathogenesis and immune responses to infection. On the other hand, the lack of clinical manifestations after hRSV infection limits the use of this model in this field. Additionally, the natural progression of infection from the URT to the LRT taking place in humans is not observed in this species (Boukhvalova and Blanco, 2013). For laboratories looking forward to including this animal model, it must be noted that handling, maintenance, and breeding of this species are different from mice strains, demanding specific facilities and protocols. Handling might be difficult to inexperienced scientists and students, and these animals may bite if not held properly. Also, blood withdrawal and drug administration are different; the retro-orbital plexus is the best site for blood collection, which requires training of personal for ensuring animal welfare (Niewiesk and Prince, 2002).

### Other Rodent Models

Beyond inbred mice and cotton rats, several other rodents have been used for hRSV studies, including vaccine efficacy studies. However, to date, robust and consistent data from established research lines are lacking, and the application of these studies to vaccine development and testing is very limited. The use of these other rodent models in general and the scope of the findings are limited by the disadvantages of being not fully permissive for hRSV replication, the use of outbred animals, and the lack of immunological reagents and inbred animals. However, recent observations highlight the importance of expanding the animal model repertoire.

Experimental infections of hamsters have been reported in 3-week-age hamsters inoculated with high hRSV doses (10^4.6^–10^6.5^ PFU), which allow for viral replication in URT and LRT but lacking histopathological and clinical manifestations of lung compromise (Wright et al., 1970). Remarkably, Syrian hamsters (*Mesocricetus auratus*) have been used for the development and preclinical evaluation of live attenuated recombinant human parainfluenza virus 1 vectors expressing the hRSV F protein. This attenuated vaccine has provided protection in these rodents against an IN challenge on day 30 post immunization, with a dose of 10^6^ PFU, protection that well correlated with the production of serum NAs against hRSV (Mackow et al., 2015).

Studies on Chinchillas (*Chinchilla lanigera*) have shown that this species is semi-permissive for hRSV infection, but only in the upper airways following high-dose intranasal challenge (1x10^6^ – 1x10^7^ PFU). It has been proposed as a suitable model for studies of hRSV infection in upper airways, including the role of hRSV infection in the development of otitis media, based on observations of viral replication in nasal cavities and Eustachian tubes (Gitiban et al., 2005; Grieves et al., 2010), as well as a model for the understanding of mucosal immunity responses (McGillivary et al., 2013); however, this model has not been employed enough to draw robust conclusions of its application.

Guinea pig (*Cavia porcellus*) is another rodent that is semi-permissible for hRSV infection, as observed in a single study to date. Acute bronchiolitis, including bronchiolar epithelial necrosis, and either mononuclear and polymorphonuclear (PMN) lymphocyte infiltrates, have been observed at 6 days post-infection, with a remission at 14 days post-infection, in one-month age outbred guinea pigs, intranasally infected with 4 × 10^3^ PFU. This was accompanied by the absence of clinical signs. The histopathological lesions at 6 days post-infection were paralleled with low viral titers, indicative of limited viral replication (Hegele et al., 1993). Although these observations support the use of this model for the understanding of acute hRSV infections, it has not been extensively studied, and the very limited availability of inbred strains and immunological reagents restricts its use. Noteworthy, an advantage on the use of this model is that guinea pig is more similar to humans than other small animal models in terms of physiology and immune system responses, which has been observed in several studies reviewed elsewhere (Padilla-Carlin et al., 2008).

## Mustelid Model

Ferrets (*Mustela putorius furo*) are small carnivores that belong to the *Mustelidae* family, which have been used for the study of different infectious diseases, including those hRSV infection. Their small size, and similarities shared with humans at an anatomical and physiopathological level are key advantages of the use of this species for investigation of infectious diseases.

In 1976, it was shown that ferrets allow for viral replication in nasal tissue of all age animals (Prince and Porter, 1976), but replication in lung tissue was observed only in infant ferrets; therefore, it was proposed as an interesting model for the age dependence studies on severe hRSV infection (Byrd and Prince, 1997). However, a recent study further highlighted the advantages of ferret models, showing that adult ferrets are highly susceptible to hRSV infection, since IT infection of immunocompetent individuals resulted in productive virus replication in the upper and lower respiratory tract, with the presence of viral antigens in tracheal and bronchial epithelial cells, reaching viral loads in throat, trachea, and lungs with similar order of magnitude in comparison to cotton rats (Stittelaar et al., 2016). This seems to be a major advantage of this model, along with the ease of handling the animals. Interestingly, recent viral interference studies have been carried out in ferrets to elucidate mechanisms that explain separate seasonal peak incidence of influenza and hRSV, showing that replication of a pandemic influenza virus strain (A[H1N1]pdm09) prevents subsequent infection with laboratory hRSV strains by antigen-independent mechanisms (Chan et al., 2018). Besides, studies on immunocompromised ferrets have shown hRSV replication in bronchiolar epithelial cells and reduced/delayed viral clearance (de Waal et al., 2018). This would allow the development of a specific animal model for a certain population at risk, such as immunocompromised patients, who experience severe pulmonary manifestations after hRSV infection (de Waal et al., 2018). Remarkably, viral transmission studies in immunocompetent or immunocompromised animals have been recently carried out, which suggest the suitability of ferrets in studying interventions aimed to limit hRSV transmission (Chan et al., 2017; de Waal et al., 2018).

Some disadvantages regarding the use of this model include the lack of clinical manifestations, the requirement of a more specialized husbandry facility and caging system, and the limited availability of immunological reagents and genetically modified mutants for immunological investigation. Also, the limited development of inbred ferrets restricts the application for mechanistic studies (Enkirch and Von Messling, 2015; Stittelaar et al., 2016).

## Large Animals Models: Lambs

Sheep (*Ovis aries*) are susceptible to natural infection by ovine respiratory syncytial virus and bovine respiratory syncytial virus (bRSV; Masot et al., 1995, 1996). The last mentioned is responsible for severe infection in preterm lambs, with an age-dependent clearance of the virus, being this preterm lamb model a useful model of severe hRSV disease in the preterm infants (Meyerholz et al., 2004), and also in exploring disease mechanisms, therapeutic regimens, and risk factors (Derscheid and Ackermann, 2012). Lambs are also susceptible to experimental infection by high doses of hRSV, which results in URTD and LTRD (Olivier et al., 2009; Sow et al., 2011; Larios Mora et al., 2015). Intratracheal inoculation of lambs with 10^8^ PFU of the A2 strain induces a pulmonary pathology that shares similarities to pathology observed after human infant hRSV infection, including bronchiolitis with neutrophil infiltration, mild peribronchiolar interstitial neumonia. A peak of viral replication in airway epithelium occurs at 6 days post-infection, with virus clearance at 14 days post-infection (Olivier et al., 2009; Sow et al., 2011). Nebulization of 7.6 × 10^7^ PFU of Memphis 37 hRSV strain results in increased expiratory effort in lambs at 4, 6, and 8 days post-infection, a viral titer peak in bronchoalveolar lavage fluid (BALF) at 3 days post-infection and viral antigen in lung tissue, both reduced at 8 days post-infection. Further, infected animals develop histopathologic lesions including bronchitis, bronchiolitis, necrosis, and hyperplasia of epithelia, peribronchial lymphocyte infiltration, and syncytial cells, resembling those described for lambs and infants (Larios Mora et al., 2015).

Lamb respiratory tract allows for a more suitable comparison with the respiratory tract of human infants than rodent model, in terms of development, structure, susceptibility to hRSV strains, pathology, immune response, and clinical manifestations (Plopper et al., 1983; Lindgren et al., 1996; Derscheid and Ackermann, 2012). Similar developmental and structural features include the size of the nasal cavity and airways, the presence of airway submucosal glands that express lactoperoxidase, prenatal alveolar development, airway branching patterns, percentage of Club cells, and development of type II cell (Scheerlinck et al., 2008; Derscheid and Ackermann, 2012; Ackermann, 2014). Additionally, lamb model offers the possibility to evaluate pulmonary functions using the same principles and techniques that are applicable for children and adults (Kirschvink and Reinhold, 2008). These advantages, along with the reported findings, establish the preterm and neonatal lamb as a model with key features that mimics hRSV infection in preterm and neonatal infants.

Additionally, induction of resistance to hRSV infection and development of hRSV-specific antibodies by neonatal and maternal immunization has been recently studied in the sheep model. Immunization of neonates with hRSV F protein in adjuvant provides effective immunization even in the presence of maternal antibodies (MatAbs; Garg et al., 2015). Moreover, pregnant ewes immunized with the same vaccine resulted in transfer of MatAbs to the newborn lambs through the colostrum. Those newborn lambs receiving MatAbs challenged with hRSV at 3 days of age showed a reduction of 70% in viral load, less lung pathology, and higher virus neutralizing titers (VNTs) than in control animals without passive immunization. This newborn lamb model of hRSV is therefore suitable for studies on maternal immunization and evaluation of the ability and safety of a vaccine to induce MatAbs and protection following hRSV challenge (Garg et al., 2016).

Studies on FI-RSV-vaccinated lambs revealed that the immune response triggered by this immunization reduced RSV titers in bronchoalveolar lavage fluids and lungs, as well as histopathology scores, but increased peribronchiolar and perivascular lymphocyte infiltration as compared to lambs undergoing either an acute RSV infection or naïve controls. Furthermore, no evident disease exacerbation was observed (Derscheid et al., 2013). These observations suggest that the lamb FI-RSV infection differs from what has been observed in children presenting FI-RSV enhanced disease (Kim et al., 1969; Prince et al., 2001). However, further studies need to be performed to understand the altered response of lambs to FI-RSV vaccination and to establish the scope of this model.

Technical advantages regarding the use of the lamb model include the ease of repeated sampling and manipulation, a long lifespan that facilitates realization and interpretation of studies on elder population. Unfortunately, there are some significant disadvantages regarding the use of the lamb model, including limited availability of molecular tools for immunologic and genetic studies, and additional efforts and requirements in housing and handling of this species (Bem et al., 2011; Gerdts et al., 2015).

## Natural Host Pneumovirus Infections

Pneumoviruses (family *Paramyxoviridae*, subfamily *Pneumovirinae*) include several pathogens of veterinary concern, including avian metapneumovirus, bovine respiratory syncytial virus (bRSV), ovine and caprine RSVs, pneumonia virus of mice (PVM), and canine pneumovirus (Easton et al., 2004; Renshaw et al., 2011). A pneumoviral infection in its natural host is associated with a fully permissive replication and a persistent inflammatory response. Interest in using some of those natural host pneumoviral infections as a model of hRSV infection has increased, since this approach of study offers several advantages over less permissive models, which are discussed later.

### bRSV in Calves

The bRSV infection in cattle (*Bos primigenius taurus*) occurs naturally and is one of the main pathogens causing pneumonia in the cattle industry along with other viral agents such as parainfluenza type 3. In addition, bRSV infection predisposes animals to secondary bacterial infections with agents such as *Mannheimia haemolytica, Pasteurella multocida, Histophilus somni,* and *Mycoplasma bovis,* producing a synergism between viral and bacterial agents known as bovine respiratory disease (van der Sluijs et al., 2010; Ellis, 2017). Therefore, bRSV infection has been extensively studied with the objective of reducing its major health and economic burden. Vaccines for bRSV are commercially available, showing variable efficacy in disease reduction (Bem et al., 2011; Ellis, 2017). Cattle can also be experimentally infected by hRSV passaged in bovine cell lines (Thomas et al., 1984). Interestingly, antigenic similarities, epidemiology, and viral pathogenesis have increased interest in studying bRSV infection in calves as an animal model of hRSV infection. Calves reproduce many of the clinical signs associated with hRSV infection in human infants, with clinical signs such as fever, rhinorrhea, cough, serous or mucopurulent nasal discharge, abnormal pulmonary auscultation, tachypnea, and hypoxia (Sacco et al., 2012a, 2015; Taylor, 2013). After bRSV infection, an incubation period of 2–5 days is followed by a range of clinical signs, from asymptomatic to URTD and severe LTRD. The severity of disease is age dependent; most severe disease occurs in young calves in the first 1–6 months of life; however, older animals are also susceptible when subjected to stressful conditions (Elvander, 1996; Hägglund and Valarcher, 2016). Experimental infection of calves results in macroscopic and microscopic lesions resembling natural infection. Lung pathology peaks at 7–8 days post-infection and includes lung consolidation, atelectasis, and sporadic deposition of hyaline membranes, with microscopic lesions of bronchointerstitial pneumonia, epithelial necrosis, alveolitis, occasional syncytial cells, debris obstruction of lumen of the bronchi and bronchioles, and extensive apoptosis of lung epithelial cells (van der Poel et al., 1996; Woolums et al., 1999; Valarcher and Taylor, 2007; Blodorn et al., 2015).

Increased levels of pro-inflammatory cytokines and chemokines, including CCL5, CCL3, IL-8, IL-6, IL-12, TNF-α, and IFN-γ, are observed after bRSV infection, as summarized from *in vitro* studies on dendritic cells (Werling et al., 2002) and *in vivo* calf studies that analyzed lung samples (Antonis et al., 2010; Sacco et al., 2012b). Calves recovering from bRSV infection demonstrate significant neutrophil accumulation in the BALF – as seen in children with hRSV bronchiolitis – as well as a predominance of CD8^+^ T cells (Taylor et al., 1989; McInnes et al., 1999; Antonis et al., 2010). Neutrophils have a role in clearance of virus that is excreted to the lumen of the respiratory tract (Viuff et al., 2002). On the other hand, recent proteomic analyses performed in bRSV-infected calves support an immunopathologic role for neutrophils in RSV disease, demonstrating a positive correlation between disease severity and neutrophil presence in alveolar septa, as well as upregulation of neutrophil-associated proteins in the BALF during infection. Increased neutrophil degranulation and diminished anti-oxidant activity were also observed in calves with bRSV disease. Noteworthy, several histones were associated with the disease, and citrullinated histone 3, an indicator of neutrophil extracellular traps (NET), was only present in unimmunized, infected animals (Hägglund et al., 2017). Considering previous studies that identified NET formation in the lungs and airways of RSV infected children presenting with severe LTRD (Cortjens et al., 2016), these results suggest that dysregulated neutrophil responses and NET activity contribute to severe immunopathology leading to LTRD after RSV infection (Afonso et al., 2016; Hägglund et al., 2017) and evidence the advantages of using a bovine model to study neutrophil-related pathological mechanisms.

Results from several studies suggest that cellular responses are crucial for clearance of bRSV. CD8^+^ T cells are the predominant lymphocyte subpopulation in the respiratory tract of calves recovering from bRSV infection (McInnes et al., 1999), and their appearance in the lungs on days 7–10 post-infection coincides with viral clearance (Gaddum et al., 1996; McInnes et al., 1999). Additionally, CD8^+^ depletion in calves results in increased severity of pneumonic consolidation and prolonged nasopharyngeal excretion of the virus (Taylor et al., 1995). Interestingly, however, rapid viral clearance has been observed in bRSV-infected calves that might be related to apoptotic mechanisms rather than T cell cytotoxicity. Apoptosis of the bronchial epithelium and phagocytosis of bRSV antigen-containing apoptotic cells were evident in the lungs at 6 days post-infection, prior to a significant recruitment of CD8^+^ cells (Viuff et al., 2002).

γδ T cells have been implicated in disease pathogenesis and the development of airway hyperreactivity following hRSV infection in humans and rodent models (Aoyagi et al., 2003; Dodd et al., 2009; Huang et al., 2015); however, it has been difficult to study their role in the disease due to their rare frequency in circulation. By contrast, γδ T cells comprise around 40% of the circulating lymphocyte pool in ruminants (Mackay and Hein, 1989), making the bovine an important model for studying the role of non-conventional T cells in bRSV infection. Bovine γδ T cells produce multiple proinflammatory cytokines and chemokines in response to *in vitro* and *in vivo* bRSV infection (McGill et al., 2013) and are a major source of IL-17 in *in vitro* bRSV antigen recall assays (McGill et al., 2016). However, their importance in this infection remains unclear, as depletion of γδ T cells does not significantly alter the course of bRSV disease (Taylor et al., 1995).

The calf model is useful for the evaluation of several aspects of vaccine development. The calf is scalable in size to human infants and is a tractable model of the neonatal immune system. Furthermore, vaccine development for bRSV faces many of the same challenges as vaccine development for hRSV, including the need to immunize infant populations and to induce long-lived and balanced immune responses, often in the face of maternal or preexisting immunity. The calf is also useful for studying vaccine-enhanced respiratory disease, as this phenomenon has been observed following natural bRSV infection of Belgian White Blue calves 3–4 months after vaccination, with fatal outcome (Kimman et al., 1989). Studies designed to experimentally induce FI-RSV enhanced respiratory disease in calves have shown mixed results regarding the onset and development of pulmonary disease (West et al., 1999; Antonis et al., 2003; Kalina et al., 2004). The differences in disease presentation may be attributable to different vaccine antigen doses, levels of serum antibodies at the time of vaccination, and the time of subsequent challenge (Taylor, 2017).

Several vaccines have been developed and tested in calves, including recombinant bRSV vaccines, modified live viruses, DNA and subunit vaccines, immunostimulating complexes, and novel adjuvants, such as CpG oligodeoxynucleotides (Oumouna et al., 2005; Letellier et al., 2008; Ellis et al., 2010; Riffault et al., 2010; Hagglund et al., 2011; Kavanagh et al., 2013; Woolums et al., 2013; Blodorn et al., 2014; Taylor et al., 2014, 2015). The calf model has also been used in preclinical safety and immunogenicity studies for vaccine candidates containing conserved proteins between both viruses, leading to phase I clinical trials in healthy human adults (Green et al., 2015; Taylor et al., 2015). The recent description of the pre-fusion form of the hRSV F protein has changed our understanding of hRSV antigenicity (McLellan et al., 2013a,b). Similarly, the recent description of the pre-fusion form of the F protein of bRSV (Zhang et al., 2017) has led to major advancements in bRSV vaccine development. Immunization of seronegative calves with a recombinant pre-fusion F protein in Montanide, an oil-in-water adjuvant, induces neutralizing antibody responses nearly 100-fold greater than similar vaccination with post-fusion form of the F protein and induces sterilizing immunity to bRSV challenge (Zhang et al., 2017). Vaccination with recombinant pre-fusion F was also recently shown to enhance neutralizing antibody responses in adult, seropositive cows (Steff et al., 2017).

Recent studies have also shown that the bovine RSV model constitutes a recommendable *in vivo* system to study therapeutic compounds aimed to control hRSV replication and airway obstruction. It has been reported that administration of GS1, a fusion inhibitor, to infected calves, provides therapeutic improvement in clinical manifestations, as well as attenuation of lung pathology and viral load (Jordan et al., 2015). More recently, Cortjens and collaborators have demonstrated that therapeutic administration of local dornase alfa reduces NET formation and airway occlusion in 4-week-old bRSV infected calves, highlighting the relevance of targeting NETs to treat severe airway obstruction induced by bRSV (Cortjens et al., 2018). Housing and handling of cattle require more expertise and specialized housing facilities; however, for veterinary personnel accustomed to working with large animals, this model has the advantage of offering a rapid and repeated sampling of the URT, in comparison to mice. Similarities in bovine and human palatine and nasopharyngeal tonsils (Rebelatto et al., 2000) provide an additional sampling zone for cattle and mark a difference from rodent models, which lack this tissue (Velin et al., 1997). Airway submucosal glands are common in humans and cattle. It has been suggested that inflammatory processes in lung traduce in only a brief detrimental effect in mice, because of the relatively large airway lumen and lack of mucosal gland (Irvin and Bates, 2003). Studies on lung function are more easily performed in calves, and the similarities in structure between calves and humans permit a more credible interpretation of those tests (Kirschvink and Reinhold, 2008). These features, added to the natural replication of the virus and clinical manifestations in calves, make this model highly relevant for studies of clinical lung disease by hRSV. The availability of reagents and tools for immunological, genetic, and molecular studies in cattle is reduced when compared to mice. However, an important number of immunological reagents, including monoclonal antibodies to cell surface molecules, recombinant cytokines and chemokines, and cross-reactive anti-human mAbs, is currently available for the cattle (Sacco et al., 2015).

### PVM in Mice

The pneumonia virus of mice (PVM) was first detected in mouse lungs (Horsfall and Hahn, 1940). This virus usually causes natural infections in mouse colonies, especially in immunodeficient mice. In immunocompetent mice, infections are short lived and mostly asymptomatic (Homberger and Thomann, 1994).

Previously described as a rodent-specific pathogen (Parker and Richter, 1982), its host range remains largely unknown, but has been reported as a cause of disease in hedgehogs (Madarame et al., 2014). PVM is the only pneumovirus that is full permissible in the rodent model, with experimental low dose infections leading to clinical disease (Cook et al., 1998; Bonville et al., 2006). C57BL/6 mice show a higher resistance to infection than BALB/c mice when challenged with lethal and sub-lethal doses, probably due to the increased ability of C57BL/6 mice to control viral replication and the immune response elicited by PVM (Watkiss et al., 2013). However, higher protective responses have been observed in a second infection challenge in C57BL/6 mice, several weeks after a previous IN inoculation with 300 PFU of PVM-15 strain (Shrivastava et al., 2015). This protective response appears to be related to an innate pro-inflammatory response and IgA in the lungs after the first inoculation, leading to a higher capacity of generating VNT during the second challenge (Shrivastava et al., 2015). Virus replication capacity is high and occurs in alveolar and bronchial epithelial cells. BALF analyses post-infection have shown an increase in eosinophil and neutrophil infiltration that progresses to an inflammatory state with almost 100% of neutrophil component (Domachowske et al., 2000). This inflammatory state induces local production of proinflammatory mediators including CCL3, CXCL2, and CCL2 (Bonville et al., 2006) consistent with those detected in lung and nasal washes in severe hRSV disease in human infants (Domachowske et al., 2004). High morbidity and mortality are associated to this infection, with clinical signs including difficult breathing, and cyanosis. Histopathological analyses show alveolar epithelial cell apoptosis, bronchial, epithelial necrosis, multifocal acute alveolitis, intraalveolar edema, multifocal hemorrhage, and granulocytic infiltration (Cook et al., 1998). A correlation between pathologic abnormalities and clinical signs has been reported with an IN inoculation of 120 PFU, showing a virus clearance at 10 days post-infection (Cook et al., 1998). A CD8^+^ T cell response with high levels of TNF-α and IFN-γ in a cytokine storm context has a major role in the pathology induced by this infection, as it has been described in C57BL/6 mice at 5 days post-infection (Walsh et al., 2014). The role of these cells in virus clearance and lung pathology was previously seen in other models of hRSV infection in mice (Cannon et al., 1988; Munoz et al., 1991). However, there are clear differences between PVM and RSV models; acute inflammatory responses to PVM infection vary in anage-dependent form, with scarce recruitment of PMN cells and inflammatory mediators in neonatal mice when compared to 3- or 4-week-age mice (Bonville et al., 2010).

Human respiratory syncytial virus vaccine development studies have been made on this model (Maunder et al., 2015; Martinez et al., 2016) including a model of FI-PVM enhanced respiratory disease, mediated by a Th2-biased response and eosinophil infiltration in lung (Percopo et al., 2009). Most recently, a replication deficient recombinant human adenovirus serotype 5 expressing the M or N protein of PVM pathogenic strain J3666, provided protection in mice, mediated mainly by CD8^+^ T cells (Maunder et al., 2015). However, there is still a limited number of studies in this field. Moreover, immunological and pathological studies are scarce too, and differences in mice and virus strain and infective doses have resulted in different clinical outcomes; therefore, drawing conclusions on this topic requires further studies. An advantage of rodent models in general is the availability of reagents and tools for immunological studies, including gene knockout mice facilitate mechanistic studies of PVM disease (Moore and Peebles, 2006) making this model useful for elucidation of inflammatory mechanisms associated with pneumovirus infection (Dyer et al., 2012).

Disadvantages of the use of this model include the high genetic distance between PVM and hRSV, which traduces in gene products with low aminoacidic sequence identity, ranging from 10 to 60%, and lack of direct cross-reactivity (Krempl et al., 2005; Dyer et al., 2012). Also, inherent to every mouse model of human pathogens, there are several differences in immune response between species and lung anatomy. Therefore, extrapolations of this model to hRSV studies should be made carefully.

## Concluding Remarks

**Figure 1:**
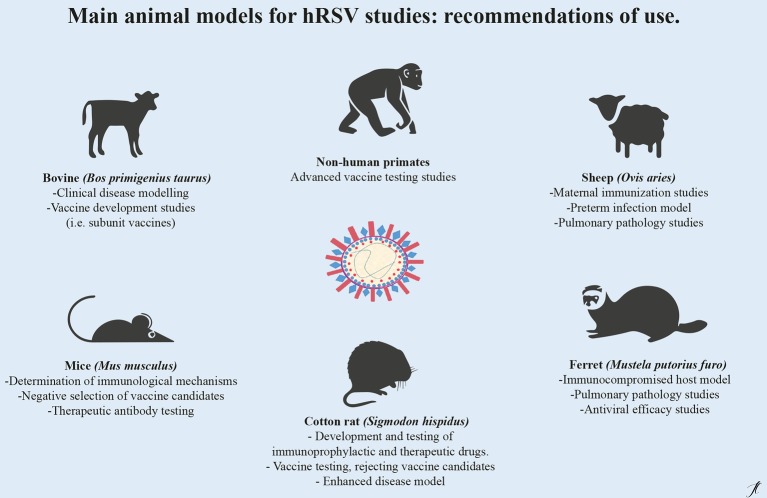
Main animal models for hRSV preclinical studies: recommendations for use. For the study of respiratory syncytial virus, there are different animal models of infection, since there is not a single animal that replicates in an integral way the human pathology and the response to treatment and vaccine candidates. The choice of the model will depend on the investigative hypothesis and should considerate the advantages and disadvantages inherent to every model.

The contribution of animal models to the study of human respiratory syncytial virus has been a fundamental part for the development of vaccines and alternative therapies. Each animal model reviewed in this document presents advantages or disadvantages (Table 1) highlighting the importance of the correct choice of the model according to the hypothesis to be evaluated or the aspect of infection that requires investigation (Figure 1).

**Table 1:** Advantages and disadvantages of different animal species suitable for modeling different aspects of hRSV-induced disease.

Animal models	Advantages	Disadvantages
Natural host pneumovirus models		
Cattle – bRSV	- Permissive model. - Manifest respiratory signs and symptoms. - Reliable lung function testing and interpretation due to anatomical similarities. - Viral antigenic similarities.	- Heterologous model. - Limited availability of molecular tools for immunologic and genetic studies. - Requires large space and specialized veterinary maintenance. - Frequent co-infections naturally occurring.
Mice – PVM	- Permissive model. - Inbred strains and transgenic lines available. - Small size and low cost of maintenance.	- Heterologous model and viral antigenic differences. - Anatomical and immunological differences. - Requirement of special biosafety facilities.
**Heterologous host hRSV models**		
Mice	- Several inbred strains and transgenic lines available. - Small size, ease of handling and housing, and low cost of maintenance. - Proper sample size easy to achieve. - Extensive laboratory use, allowing for results comparison.	- Lung anatomical differences. - Several immune system differences. - Moderate natural viral replication.
Cotton rats	- More permissive than other models. - More immunological similarities to humans than inbred mice. - Increasing genetic and immunological molecular tools available. - Inbreed animals commercially available.	- Requires expert handling. - Less availability of molecular tools for immunologic and genetic studies, when compared to inbred mice. - Lack of transgenic lines. - Lack of clinical signs resembling human disease.
Ferrets	- Easy handling and sampling. - High susceptibility to infection.	- Lack of clinical disease. - Requires specialized housing, caring, and maintenance. - Limited availability of molecular tools for immunologic and genetic studies. - Lack of inbreed animals commercially available.
Lambs	- Reliable lung function testing and interpretation due to anatomical similarities. - Large size and docility allow for repeated and easy sampling.	- Requires specialized housing, caring, and maintenance. - Limited availability of molecular tools for immunologic and genetic studies.
Chimpanzees	- Permissive replication of hRSV - Anatomical, physiological, and genetic similarities to humans.	- High economical cost, logistically demanding. - Strong ethical and emotional compromise. - Lack of inbred strains, few available immunological tools, and reagents. - Requires extensive caring and highly specialized housing.

Within the complexity of the natural development of hRSV infection, we emphazise that there is no animal model that recapitulates morbidity and mortality trends, since high viral doses are needed to establish infection and/or manifest disease, as is the case of mice or cotton rats. Among other models of hRSV infection, we highlight the potential of ruminant models to understand pathology aspects of hRSV infection, since calves display a physiopathology very similar to infected human infants, showing very similar histopathological lesions. In case of non-human primates, it is noteworthy that they allow for viral replication with doses lower than those used in other models such as rodents, being a more permissive model for the study of hRSV. However, its use should be restricted exclusively to vaccine testing studies, due to ethical and technical issues. The antecedents reviewed here lead us to conclude that each animal model for the study of hRSV is fundamental for the understanding of the viral pathogenesis and all of them have to a greater or lesser extent to the development of biomedical tools against this agent. Expanding the availability of sophisticated models might be crucial to overcome information gaps and developing safe, effective therapies such as monoclonal antibodies or vaccines. To date, within all existing preclinical models, the murine model is the first preclinical approach for the development of new drugs, being a versatile tool that allows sophisticated immunological studies. Even considering its limitations, this model allow us to advance towards in other animal models that have more complex and expensive requirements in their management, maintenance, and infrastructure, as described in this work.

## Author Contributions

All authors listed have made a substantial, direct and intellectual contribution to the work, and approved it for publication.

### Conflict of Interest Statement

The authors declare that the research was conducted in the absence of any commercial or financial relationships that could be construed as a potential conflict of interest.
